# Regular use of proton-pump inhibitors and risk of stroke: a population-based cohort study and meta-analysis of randomized-controlled trials

**DOI:** 10.1186/s12916-021-02180-5

**Published:** 2021-12-03

**Authors:** Man Yang, Qiangsheng He, Fang Gao, Krish Nirantharakumar, Tonny Veenith, Xiwen Qin, Amy T. Page, Martin C. S. Wong, Junjie Huang, Zi Chong Kuo, Bin Xia, Changhua Zhang, Yulong He, Wenbo Meng, Jinqiu Yuan, Yihang Pan

**Affiliations:** 1 The First Clinical Medical School of Lanzhou University, Lanzhou, Gansu China; 2 Guangdong Provincial Key Laboratory of Gastroenterology, Center for Digestive Disease, The Seventh Affiliated Hospital, Sun Yat-sen University, Shenzhen, 518107 Guangdong China; 3 Department of General Surgery, The First Hospital of Lanzhou University, Lanzhou, Gansu China; 4 Big Data Center, The Seventh Affiliated Hospital, Sun Yat-sen University, Shenzhen, 518107 Guangdong China; 5 Clinical Research Center, The Seventh Affiliated Hospital, Sun Yat-sen University, Shenzhen, 518107 Guangdong China; 6 Birmingham Acute Care Research Group, Institute of Inflammation and Ageing, University of Birmingham, Birmingham, UK; 7 Institute of Applied Health Research, University of Birmingham, Edgbaston, B15 2TT Birmingham, UK; 8 Centre for Medicine Use and Safety, Faculty of Pharmacy and Pharmaceutical Science, Monash University, Melbourne, Australia; 9 School of Population and Global Health, Faculty of Medicine, Density and Health Sciences, University of Western Australia, Perth, Australia; 10 Jockey Club School of Public Health and Primary Care, The Chinese University of Hong Kong, Shatin, Hong Kong

**Keywords:** Proton pump inhibitor, Stroke, Cohort, Meta-analysis, Randomized control trial

## Abstract

**Background:**

Although randomized controlled trials (RCTs) have suggested a non-significant increased risk of stroke among proton pump inhibitor (PPI) users, the association has not been confirmed. We evaluated the association between regular use of PPIs and incident stroke and identified population groups at high net risk.

**Methods:**

This is a prospective analysis of 492,479 participants free of stroke from the UK biobank. Incident stroke was identified through linkage to hospital admission and death registries using the International Classification of Diseases (ICD)-10 codes (I60, I61, I63, and I64). We evaluated hazard ratios (HRs) adjusting for demographic factors, lifestyle habits, prevalent comorbidities, concomitant use of medications, and indications of PPIs. We assessed the risk differences (RDs) according to the baseline Framingham Stroke Risk Score. In the meta-analysis, we searched PubMed, EMBASE, and the Cochrane Central Register of Controlled Trials (from 1988 to 1 June 2020) for randomized trials comparing PPIs with other interventions, placebo, or no treatment on stroke risk. Results were combined using a fix-effect meta-analysis (Mantel-Haenszel method).

**Results:**

We documented 5182 incident strokes over 3,935,030 person-years of follow-up. Regular PPI users had a 16% higher risk of stroke than non-users (HR 1.16, 95% CI 1.06 to 1.27). The estimated effect was similar to our meta-analysis of nine RCTs (case/participants 371/26,642; RR 1.22, 95% CI 1.00 to 1.50; quality of evidence: moderate). The absolute effect of PPI use on stroke increased with the baseline Framingham Stroke Risk Score, with an RD of 1.34‰, 3.32‰, 4.83‰, and 6.28‰ over 5 years for the lowest, quartile 2, quartile 3, and the highest quartile, respectively.

**Conclusions:**

Regular use of PPIs was associated with an increased risk of stroke, with a higher absolute risk observed in individuals with high baseline stroke risk. Physicians should therefore exercise caution when prescribing PPIs. An assessment of the underlying stoke risk is recommended for individualized use of PPIs.

**Supplementary Information:**

The online version contains supplementary material available at 10.1186/s12916-021-02180-5.

## Background

Proton pump inhibitors (PPIs) are among the most frequently prescribed drugs [1], widely used to treat gastroesophageal reflux disease (GERD), peptic ulcer, upper gastrointestinal bleeding, and other acid-related disorders [2]. Although short-term use of PPIs is generally safe, accumulating evidence has linked long-term PPI use to various adverse effects such as bone fractures, chronic kidney disease, type 2 diabetes, rheumatoid arthritis, and cancer [2–8]. Concerns have also been raised over the increased risk of stroke in PPI users, particularly for patients with concomitant use of antiplatelet agents [9–13].

Although a number of studies have investigated PPI use and risk of stroke [9–14], the relationship remained unclear. First, findings of published studies were inconsistent, showing either a positive [9–13] or a null association [14–16]. Second, the existing evidence showing an association between PPIs and stroke were observational studies. Importantly, these studies were limited by either inadequate assessment of exposures and outcomes through retrospective recall or administrative claims or insufficient adjustment of important confounders such as lifestyle habits and indications of PPI therapies [9–13, 15, 16]. Third, randomized controlled trials (RCTs) may provide the highest level of evidence; however, current RCTs were underpowered to detect the effect of PPIs on stroke, although many trials have demonstrated an association towards increased risk [17–19]. For example, a recent RCT including over 17,000 participants found that pantoprazole appeared to have a modest, although not statistically significant, increased risk of stroke when compared with placebo (hazard ratio [HR], 1.16; 95% confidence interval [CI], 0.94 to 1.44) [19]. There have also been no secondary studies conducted to combine these RCTs. Lastly, the investigation of subgroups at the high absolute risk of stroke among PPI users is still lacking. It has been shown that the absolute effects (often presented with risk difference [RD]) of interventions tended to increase with the baseline risk [20]. Thus, individualized treatment based on patients’ underlying risk may confer benefits and reduce harms. Such risk stratification strategy has been applied to select patients for antihypertensive and statin therapy [21, 22]. Similarly, risk stratification may potentially be applied to guide the individualized use of PPIs. For those without increased absolute risk, PPIs could be safely used. While for those at high risk, stopping or replacing PPIs, in conjunction with regular screening for stroke might be necessary.

In the present study, we conducted a prospective analysis of the UK Biobank cohort and a meta-analysis of RCTs to (1) evaluate the association between PPI use and subsequent risk of stroke and (2) investigate which population groups may have a high net risk of stroke associated with PPI use.

## Methods

### Population-based prospective cohort study

#### Study population

UK Biobank is a large-scale, long-term prospective study containing in-depth genetic and health information from half a million UK participants. Between 2006 and 2010, UK biobank enrolled 502,528 participants aged 37–73 years from 21 assessment centers across England, Wales, and Scotland. At recruitment, with their consent participants visited the closest assessment center to provide blood, urine, and saliva samples, as well as detailed information about sociodemographic, lifestyle and health-related factors, environment and medical history via touchscreen and face-to-face interviews. A range of physical measurements, including height, body weight, and blood pressure were taken. Follow-up assessments were conducted through linkages to routinely available national datasets. More details of UK Biobank design can be found elsewhere [23, 24]. The UK Biobank cohort has been approved by the North West Multi-center Research Ethics Committee, the England and Wales Patient Information Advisory Group, and the Scottish Community Health Index Advisory Group. All participants had provided written informed consent prior to data collection. In the present study, we excluded participants with stroke diagnosis prior to baseline (*n*=8750), and those who subsequently withdrew from the study (*n*=1299), leaving a total of 492,479 participants included in this analysis.

#### Assessment of PPI use

At baseline, regular use of PPIs was firstly assessed from participants using a touchscreen questionnaire and then confirmed during verbal interviews with a trained staff. In the touchscreen questionnaire, participants were asked “Do you regularly take any prescription medications?”. “Regular use” was defined as taking the medication in most days of the week for the last 4 weeks. If the participant selected “Yes” or “Unsure,” then they would be asked by the interviewer: “In the touch screen you said you are taking regular prescription medications. Can you now tell me what these are?” Information about PPI use was recorded in free text. The recorded type of PPIs included omeprazole, lansoprazole, pantoprazole, rabeprazole and esomeprazole. Information about doses and duration of PPIs was not collected. The detailed questions regarding PPI use could be found elsewhere [24].

#### Ascertainment of stroke

Participants were followed through linkage to the Health and Social Care Information Centre (in England and Wales) and the National Health Service Central Register (in Scotland). The primary outcome of the study was the incidence of stroke, which was linked to hospital admission and death registered using the International Classification of Diseases (ICD)-10 codes (I60, I61, I63, and I64). We classified stroke as ischemic stroke (I63, I64), intracerebral hemorrhage (I61), or subarachnoid hemorrhage (I60). Details of the methods used to identify stroke could be found on the UK Biobank website [24]. At the time of analysis, complete follow-up was available up to 31 October 2017 for England and 31 October 2016 for Wales and Scotland.

#### Assessment of covariates

Covariate information was obtained at baseline. Sociodemographic factors (*age*, *sex*, *ethnicity*), lifestyle habits (*smoking status*, *alcohol consumption*, and *dietary intake*), family history of stroke, multivitamin use, and intake of mineral supplements were self-reported. Index of multiple deprivation, a composite measure of socioeconomic status, was provided directly from the UK Biobank. Physical activity was assessed using the International Physical Activity Questionnaire - Short Form (IPAQ-SF). Current concomitant comorbidities (*hypertension*, *hypercholesterolemia*, *diabetes*, *prevalent cardiovascular disease [CVD]* (*including coronary artery disease*, *congestive heart failure*, and *peripheral vascular disease*), *atrial fibrillation*, *cancer*, *esophagitis/Barretts esophagus*, *GERD*, and *peptic ulcer*), and medication use (*aspirin*, *non-aspirin non-steroidal anti-inflammatory drugs [NSAIDs]*, *acetaminophen*, *antihypertensive drugs (including angiotensin-converting enzyme inhibitors*, *angiotensin II receptor blockers*, *beta-blockers*, *calcium channel blockers*, and *thiazide diuretics)*, *statin*, *metformin*, *histamine-2 receptor antagonists [H2RAs]*, *antiplatelets*, and *clopidogrel*) were assessed based on self-reported medical history, which were subsequently verified during face-to-face interview. Height and weight were measured by trained research staff and used to calculate body mass index (BMI). More details of these measures could be found elsewhere [24].

#### Statistical analysis

We calculated person-years from the recruitment date to the date of the first diagnosis of stroke, death, or the last date of follow-up, whichever happened first. We estimated the HRs of PPI use on stroke using Cox regression models taking age as the timescale. In the basic model, we stratified the analyses jointly by sex and age (37–54, 55–64, ≥65 years). In the multivariable-adjusted model 1, we adjusted for ethnicity, socioeconomic status, smoking status, alcohol consumption, physical activity, fruit and vegetable intake, BMI, multivitamin and mineral supplements intake, family history of stroke, history of hypertension, hypercholesterolemia, diabetes, CVD, atrial fibrillation, and cancer. We additionally adjusted for medications use (including aspirin, non-aspirin NSAIDs, acetaminophen, antihypertensive drugs, statin, and metformin) in the multivariable-adjusted model 2. To address the possible confounding effect of clinical indications for PPI use, we additionally adjusted for esophagitis/Barrett’s esophagus, GERD, peptic ulcer, H2RA use, and anticoagulant/antiplatelet use in the multivariable-adjusted model 3. Proportional hazards assumption was checked using Schoenfeld’s tests and no violation was shown. For covariates with selections of “do not know” and “prefer not to answer,” or with missing data, we included an “unknown/missing” value indicator. To present the association in a clinically useful way, we calculated the number needed to harm (NNH) and RD based on the method described by Altman D.G and Andersen P.K [25].

We also evaluated the baseline stroke risk of included participants using the Framingham Stroke Risk Score [26], based on which, we stratified the participants into subgroups of different risks. Then, we evaluated the relative effect (by HR) and absolute effect (by RD) of PPIs on stroke at each subgroup. We conducted additional stratified analyses according to sex, age, BMI, smoking status, alcohol consumption, physical activity, history of hypertension, hypercholesterolemia, diabetes, regular use of aspirin, history of GERD, and any clinical indications for PPI use.

We performed a number of sensitivity analyses to check the robustness of the primary results. First, we excluded participants who developed stroke or died during the first two years of follow-up to minimize reverse causality. Second, we excluded participants with cardiovascular disease or cancer to investigate the potential influence of the medical condition. Third, to evaluate potential bias from unobserved patient or physician characteristics (i.e., physicians may be more likely to prescribe PPIs to the patients with more severe underlying illness and also may be more likely to diagnose their patients with stroke in the appropriate clinical setting) [27, 28], we adjusted the number of self-reported operations, number of self-reported cancers, and number of self-reported non-cancer illnesses as surrogate indicators. Forth, we restricted the analyses to participants with no missing data on any covariates. Fifth, we calculated a propensity score for the likelihood of PPIs by multivariate logistic regression conditional on aforementioned baseline covariates. Then we applied inverse treatment probability weights based on the propensity scores, which creates a weighted pseudo cohort where treatment assignment is independent of measured confounders. To verify if potential biases could have modified the association between PPI use and stroke, we used falsification analyses for negative control outcomes (malignant melanoma cancer and transportation-related death) with the method described by Lipsitch M [29, 30]. We assumed that there should be no associations between PPI use and negative control outcomes. If these associations exist, the association between PPI use and stroke may be due to potential biases. We performed the analyses using SAS software, version 9.4 (SAS Institute, Cary, NC, USA).

### Meta-analysis

#### Literature search

We searched PubMed, EMBASE, and The Cochrane Central Register of Controlled Trials (CENTRAL, in The Cochrane Library) (from 1988 to 1 June 2020) for eligible studies, with no restriction in publication status and language. The search strategy was developed by an experienced group member (Jinqiu Yuan) and checked by two researchers from other teams (Zuyao Yang, The Chinese University of Hong Kong, China; Hongtao Wang, The Fourth Military Medical University, China) according to the PRESS 2015 Guideline Evidence-Based Checklist [31]. The search strategy included terms for PPIs and a sensitive search strategy for randomized controlled trials, using the following combined keywords and MeSH terms: “*proton pump inhibitors*,” “*omeprazole*,” “*esomeprazole*,” “*rabeprazole*,” “*pantoprazole*,” and “*randomized controlled trials*” (see the complete search strategy for PubMed in Additional file 1: Table S1). We also searched the reference list of relevant review articles and included studies for additional eligible studies.

#### Study selection

We included RCTs comparing PPIs with other interventions, placebo, or no treatment on stroke risk. Because the incidence of stroke is low in the population and small studies are unable to provide a reliable estimate of incidence, we only included trials that reported at least one case of stroke during follow-up, with a follow-up duration ≥ 6 months, and with a sample size ≥ 100. The outcome for meta-analysis was any stroke, included ischemic stroke, intracerebral hemorrhage, and subarachnoid hemorrhage. Study selection was undertaken by two authors (Man Yang and Qiangsheng He). We excluded trials about *Helicobacter pylori* eradication for the potential influence of antibiotics. Disagreements were resolved by discussion with a third reviewer (Jinqiu Yuan).

We initially imported all search citations into the reference management software and removed duplicated citations. We evaluated the eligibility of the remaining studies by examining the titles and abstracts. The full texts of potential eligible articles were retrieved to evaluate the eligibility. When two or more papers were published from a same study and the results were inconsistent, we only included the one with the largest sample size, most updated data, and the most relevant outcomes.

***Data extraction***

Two investigators (Qiangsheng He and Man Yang) extracted data and resolved disagreements by discussion. We extracted data with a pre-designed form for this study. The data extracted included study characteristics, methodological information, participant characteristics, intervention and control regimens, and outcomes. Missing outcome data were obtained by contacting authors and retrieving from clinical trial registries.

#### Assessment of risk of bias and quality of evidence

Two investigators (Qiangsheng He and Man Yang) evaluated the methodological quality of included studies using the Revised Cochrane Collaboration’s tool for assessing risk of bias (ROB 2) [32]. The strength of evidence for primary estimates was evaluated using the Grading of Recommendations Assessment, Development and Evaluation system (GRADE) [33].

#### Data-analysis

We undertook meta-analyses if included studies appeared appropriately similar in terms of patient population, intervention type, and outcome assessment. The summary effect size was measured as a risk ratio (RR), together with its 95% confidence interval (CI). We evaluated statistical heterogeneity with the *Q*-test and the *I*^2^ -index statistic. We carried out a meta-analysis with a fix-effect model (Mantel-Haenszel method). We evaluated publication bias with funnel plots and Egger’s test. We undertook sensitivity analyses to check the robustness of the primary result: (1) excluding studies with high risk of bias in one or more domains; (2) we excluded the COMPASS study which took up 94.3% weighting in the primary analysis. Meta-analyses were performed with Review Manager (Version 5.3. Copenhagen: Nordic Cochrane Centre, The Cochrane Collaboration, 2014).

## Results

Table 1 showed the baseline characteristics of the study participants by PPI use. At baseline, 49,135 (9.98%) participants reported regular use of PPIs, of whom 31 898 used omeprazole, 17,227 used lansoprazole, 2376 used esomeprazole, 1119 used rabeprazole, and 951 used pantoprazole. Compared with non-PPI users, regular users tended to be less physically active, with higher BMI, consumed less alcohol, with a higher prevalence of hypertension, hypercholesterolemia, CVD, diabetes, atrial fibrillation, and were more likely to use other medications (aspirin, paracetamol, metformin, and statin). As expected, PPI users had a higher prevalence of esophagitis/Barrett’s esophagus, GERD, peptic ulcer, and anticoagulants/antiplatelet treatments.

**Table 1:** Baseline characteristics of participants by proton pump inhibitor use in the UK Biobank

	Regular PPI use		Overall
	No	Yes	
Number of participants^*^	443,344 (90.02)	49,135 (9.98)	492 479
**Mean (SD) age, years**	56.6 (8.11)	60.0 (7.25)	57.0 (8.09)
**Female**	241,809 (54.5)	27,170 (55.3)	268,979 (54.6)
**White**	419,216 (94.6)	46,772 (95.2)	465,988 (94.6)
**Mean (SD) Index of multiple deprivation**	12.5 (15.8)	14.5 (19.3)	12.7 (16.1)
**Never smoker**	248,969 (56.2)	23,179 (47.2)	272,148 (55.3)
**Alcohol consumption**			
Daily or almost daily	91,205 (20.6)	8635 (17.6)	99,840 (20.3)
One to four times a week	220,827 (49.8)	21,250 (43.2)	242,077 (49.2)
One to three times a month	49,106 (11.1)	5657 (11.5)	54,763 (11.1)
Special occasions only or never	82,206 (18.5)	13,593 (27.7)	95,799 (19.5)
**Median (IQR) physical activity, MET minutes/week**	1790 (2750)	1540 (2750)	1770 (2750)
**Mean (SD) fruit and vegetable intake**	4.63 (3.11)	4.61 (3.24)	4.63 (3.12)
**Mean (SD) BMI, kg/m**^2^	27.2 (4.69)	29.2 (5.15)	27.4 (4.78)
**Family history of stroke**	114,963 (25.9)	14,430 (29.4)	129,393 (26.3)
**Comorbidities**			
Hypertension	25,2407 (56.9)	34,957 (71.1)	287,364 (58.4)
Hyperlipidemia	217,713 (49.1)	33,552 (68.3)	251,265 (51.0)
CVD	19,327 (4.4)	7809 (15.9)	27,136 (5.5)
Diabetes	23,795 (5.4)	5710 (11.6)	29,505 (6.0)
Atrial fibrillation	5336 (1.2)	1396 (2.8)	6732 (1.4)
Cancer	22,376 (5.0)	3837 (7.8%)	26,213 (5.3%)
Esophagitis/barretts esophagus	2490 (0.6)	4303 (8.8)	6793 (1.4)
Gastroesophageal reflux disease	9331 (2.1)	19,699 (40.1)	29,030 (5.9)
Peptic ulcer	4578 (1.0)	6075 (12.4)	10,653 (2.2)
**Other drug use**			
Aspirin	55,255 (12.5)	11,298 (23.0)	66,553 (13.5)
Non-aspirin NSAIDs	71,757 (16.2)	8406 (17.1)	80,163 (16.3)
Paracetamol	93,535 (21.1)	16,269 (33.1)	109,804 (22.3)
Antihypertensive drugs	38,163 (8.6)	8418 (17.1)	46,581 (9.5)
Metformin	10,994 (2.5)	2799 (5.7)	13,793 (2.8)
Statin	60,711 (13.7)	15,428 (31.4)	76,139 (15.5)
H2RAs	7975 (1.8)	2153 (4.4)	10,128 (2.1)
Anticoagulants/antiplatelets	5375 (1.2)	2110 (4.3)	7485 (1.5)
**Multivitamin use**	65,417 (14.8)	8696 (17.7)	74,113 (15.0)
**Intake of mineral supplements**	95,168 (21.5)	10,139 (20.6)	105,307 (21.4)

*CVD*, cardiovascular disease; *H2RAs*, histamine-2 receptor antagonists; *NSAIDs*, non-steroidal anti-inflammatory drugs

^*^Values are numbers (percentages) unless stated otherwise

Over a median follow-up of 8.0 years, we identified 5182 incident strokes. The event rate among regular PPI users was 2.22/1000 person-years, compared with 1.19/1000 person-years among non-users (Table 2). In the age and sex-stratified model, regular PPI users had a 1.45-fold increased risk of stroke as compared to non-users (HR 1.45, 95% CI: 1.34 to 1.56). The association was attenuated after adjustment for sociodemographic factors, lifestyle habits, prevalent comorbidities, and concomitant use of medications (HR 1.17, 95% CI 1.08 to 1.26). The estimated HR was similar after additional adjustment for clinical indications for PPI use (HR 1.16, 95% CI 1.06 to 1.27). For ease of interpretation, we calculated NNHs based on the fully adjusted HR and the incidence rate among non-PPI users (Additional file 1: Fig. S1). Every 1274.5 (95% CI, 1002.7 to 2527.1), 677.8 (95% CI, 522.2 to 1391.4), and 300.2 (95% CI, 224.2 to 634.7) regular PPI users may result in one case of stroke over 1, 2, and 5 years, respectively. Regarding stroke subtypes, PPI use was associated with an increased risk of ischemic stroke (HR 1.16, 95%CI 1.06 to 1.27) and subarachnoid hemorrhage (HR 1.47, 95%CI 1.12 to 1.94), but not with intracerebral hemorrhage (HR 1.06, 95%CI 0.84 to 1.34).

**Table 2:** Risk of stroke by regular use of proton pump inhibitors in the UK Biobank

	Cases/person-years	Hazard ratio [95% confidence interval]			
		Age and sex-stratified model	Multivariable adjusted model 1^**†**^	Multivariable adjusted model 2^**‡**^	Multivariable adjusted model 3^**¶**^
**All stroke**					
Non-regular PPI user	4326/3,549,337	1.00 [reference]	1.00 [reference]	1.00 [reference]	1.00 [reference]
Regular PPI user	856/385 693	1.45 [1.34, 1.56]	1.17 [1.09, 1.27]	1.17 [1.08, 1.26]	1.16 [1.06, 1.27]
**Ischemic stroke**					
Non-regular PPI user	3359/3,551,582	1.00 [reference]	1.00 [reference]	1.00 [reference]	1.00 [reference]
Regular PPI user	693/386,096	1.48 [1.37, 1.61]	1.17 [1.07, 1.27]	1.15 [1.06, 1.26]	1.14 [1.04, 1.26]
**Intracerebral hemorrhage**					
Non-regular PPI user	720/3,560,115	1.00 [reference]	1.00 [reference]	1.00 [reference]	1.00 [reference]
Regular PPI user	119/387,845	1.15 [0.95, 1.40]	1.01 [0.83, 1.23]	1.01 [0.83, 1.24]	1.06 [0.84, 1.34]
**Subarachnoid hemorrhage**					
Non-regular PPI user	484/3,551,582	1.00 [reference]	1.00 [reference]	1.00 [reference]	1.00 [reference]
Regular PPI user	90/386,096	1.56 [1.24, 1.96]	1.50 [1.19, 1.89]	1.50 [1.19, 1.90]	1.47 [1.12, 1.94]

†Multivariable adjusted model 1: additionally adjusted for ethnicity (white, or other), socioeconomic status (index of multiple deprivation, fifth), smoking status (never smoker, previous smoker, or current smoker), alcohol consumption (daily or almost daily, one to four times a week, one to three times a month, special occasions only or never), physical activity (low, moderate, or high), fruit and vegetable intake (≥5 portions or < 5 portions), BMI, multivitamin use, and mineral supplements intake (yes or no), family history of stroke (yes or no), hypertension (yes or no), hypercholesterolemia (yes or no), diabetes (yes or no), prevalent cardiovascular disease (including coronary artery disease, congestive heart failure, and peripheral vascular disease, yes or no), atrial fibrillation (yes or no), cancer (yes or no)

‡Multivariable adjusted model 2: additionally adjusted for medications use, including aspirin, non-aspirin NSAIDs, acetaminophen, antihypertensive drugs (including angiotensin-converting enzyme inhibitors, angiotensin receptor blocker, beta-blockers, calcium channel blockers, and thiazide diuretics), statin, and metformin

¶Multivariable adjusted model 3: additionally adjusted for esophagitis/Barretts esophagus (yes or no), gastroesophageal reflux disease (yes or no), peptic ulcer (yes or no), histamine-2 receptor antagonists use (yes or no), and anticoagulants/antiplatelets (yes or no)

Multicollinearity assumption in the final model was checked using variance inflation factor (VIF) values and no violation was found (all VIFs < 4)

**Fig. 1:**
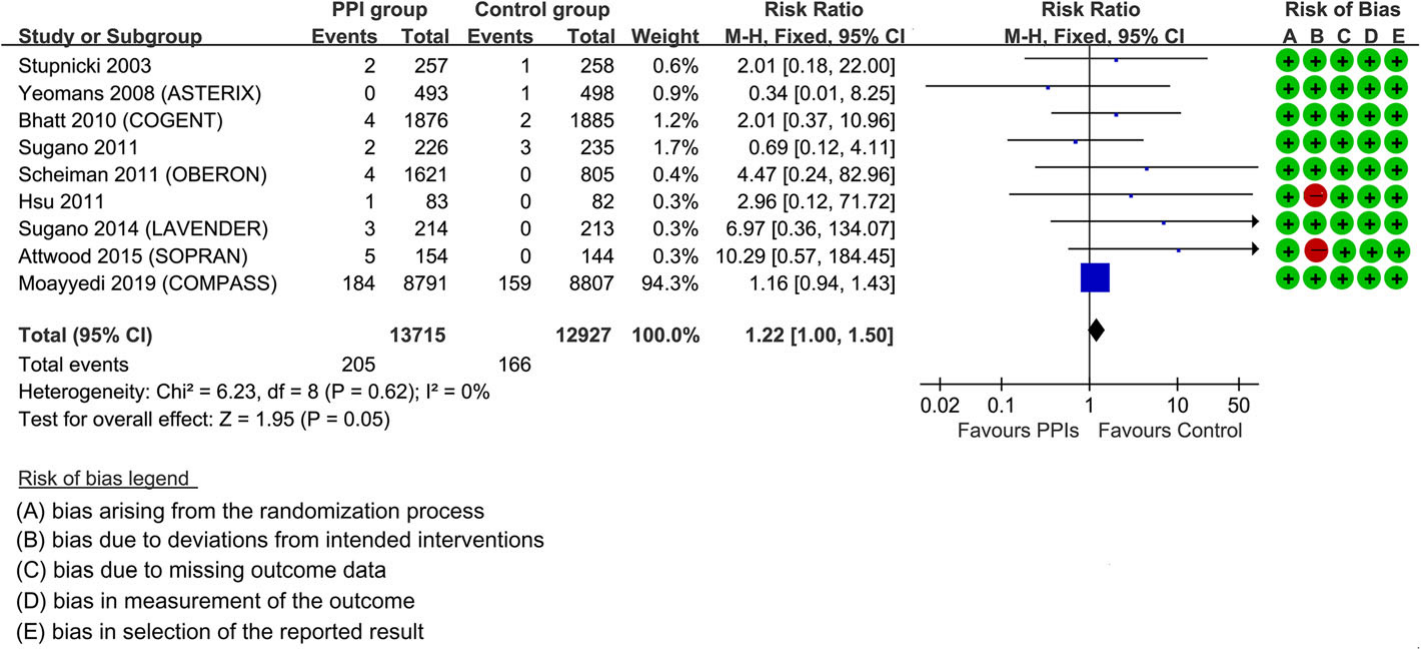
Risk of stroke by proton pump inhibitor use, meta-analysis of randomized controlled trials. Risk of bias: Two studies might have bias due to deviations from intended interventions because it is an open-labeled trial. Quality of evidence: moderate. Based on the GRADE system, this meta-analysis of randomized controlled trials was initially rated as high-quality evidence. Because two included studies were open-labeled trials, we downgraded the level of quality. There were also no other factors that may downgrade the quality in terms of the inconsistency of results, indirectness of evidence, imprecision, and reporting bias

Meta-analysis of RCTs provided the best evidence of this association. Our meta-analysis identified 13,629 potential eligible studies, of which nine trials were included [17–19, 34–39] (see the flowchart of the study selection in Additional file 1: Fig. S2). Additional file 1: Table S2 presented the baseline characteristics of the included studies. Eight trials [18, 19, 34–39] evaluated the effect of PPIs for preventing NSAID/aspirin/clopidogrel-related gastrointestinal lesions and one [17] compared omeprazole with antireflux surgery for the treatment of reflux esophagitis. There was generally no major risk of bias among the included trials except that the two trials [17, 39] were open-labeled (Fig. 1). Our meta-analysis included 371 cases and 26,642 participants. The estimated RR of stroke was 1.22 (95% CI 1.00 to 1.50; heterogeneity: *I*^2^ =0.0%, *P* = 0.62; quality of evidence: moderate), which was similar to our estimated effect from the UK Biobank. Funnel plot was generally symmetric (Egger’s test: *P* = 0.19), suggesting a low possibility of publication bias (Additional file 1: Fig. S3). Sensitivity analyses by excluding two trials [17, 39] with high risk of bias (RR 1.19, 95% CI 0.97 to 1.46; heterogeneity: *I*^2^ =0.0%, *P* = 0.71) and the COMPASS trial [19] which takes up 94.3% weighting in the primary analysis (RR 2.23, 95% CI 1.01 to 4.93; heterogeneity: *I*^2^ =0.0%, *P* = 0.56) did not change the primary result.

**Fig. 2:**
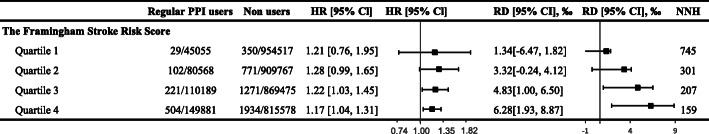
Relative and absolute effects of proton pump inhibitor use on stroke by the baseline risk. Abbreviation: CI, confidence interval; HR, hazard ratio; NNH, the number needed to harm; PPI, proton pump inhibitor; RD, risk difference; estimated HRs were based on the fully adjusted model (see the footnote in Table 2). The RDs were estimated based on the corresponding HRs and incidence rate in the non-user group, with the method described by Altman D.G and Andersen P.K

Figure 2 presented the relative and absolute effect of PPIs on stroke according to the baseline risk in the UK biobank. The relative effects were similar among subgroups, with a HR of 1.28 (95% CI, 0.99 to 1.65) in quartile 2, 1.22 (95% CI 1.03 to 1.45) in quartile 3, and 1.17 (95% CI 1.04 to 1.31) in quartile 4. We did not find sufficient evidence of an association in the lowest quartile (HR 1.21, 95% CI, 0.76 to 1.95). On the contrary, the absolute effects dramatically increased with the baseline Framingham Stroke Risk Score, with an RD of 1.34‰ (95% CI − 6.47‰ to 1.82‰) in the lowest quartile, 3.32‰ (95% CI, − 0.24‰ to 4.12‰) in quartile 2, 4.83‰ (95% CI, 1.00‰ to 6.50‰) in quartile 3, and 6.28‰ (95% CI, 1.93‰ to 8.87‰) in the highest quartile, over 5 years.

The risk of stroke for an individual class of PPIs was presented in Additional file 1: Table S3. Omeprazole was associated with an increased risk of stroke (HR 1.08, 95% CI 1.06 to 1.31). We did not find sufficient evidence of associations for other PPIs, largely due to the relatively low number of cases. We also evaluated the risk of stroke associated with regular use of H2RAs, a less profound acid suppressor with similar clinical indications as PPIs. After adjustment for potential confounders, we did not find sufficient evidence of an association between H2RA use and risk of stroke (HR 1.10, 95% CI 0.93 to 1.29).

**Fig. 3:**
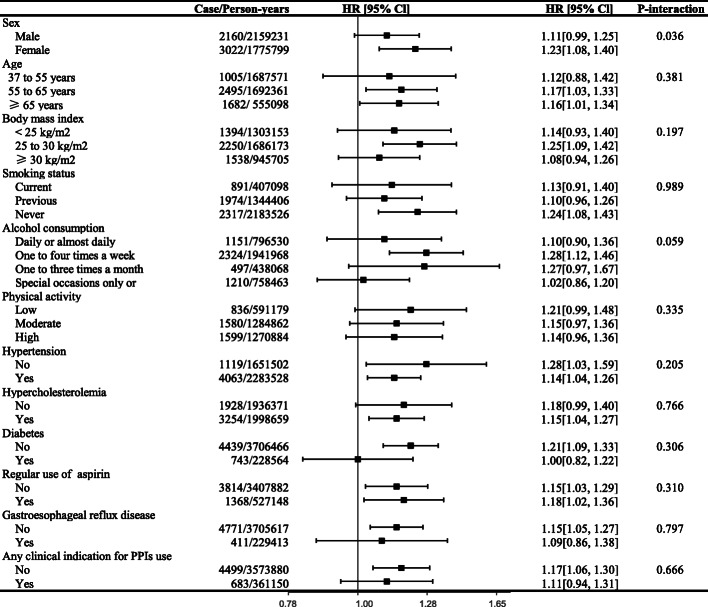
Subgroup analyses of proton pump inhibitor use and risk of stroke. Abbreviation: CI, confidence interval; HR, hazard ratio. Estimated effects were based on the fully adjusted model (see the footnote in Table 2)

Subgroup analyses showed that the estimated HRs did not differ by age, BMI, smoking status, alcohol consumption, physical activity, hypertension, hyperlipidemia, diabetes, regular use of aspirin, presence of GERD, and clinical indication for PPI use (Fig. 3 and Additional file 1: Table S4). However, the association between PPI use and the risk of stroke was slightly stronger in females than males (*P*-interaction =0.036). Sensitivity analyses by excluding the cases identified during the first 2 years of follow-up, excluding the participants with cardiovascular disease or cancer at baseline, or with missing covariate data, additionally adjustment of patient health indicators, and using propensity score analysis did not show major changes in the primary results (Additional file 1: Table S5). In the falsification analyses, regular PPI use, as expected, was not associated with risk of malignant melanoma cancer (HR 1.00, 95%CI 0.83–1.20), and death caused by transportation (HR 1.05, 95%CI 0.50–2.20) (Additional file 1: Table S6). Similar results were also observed in previous studies [4, 30].

## Discussion

This study combined the strengths of a large-scale prospective cohort study and meta-analysis of RCTs. The UK Biobank cohort, which may suffer from confounding bias, precisely estimated the effect of PPIs on stroke and investigated risk stratification; while the meta-analysis provided the highest level of evidence on this association. Our analysis of the UK Biobank demonstrated that regular PPI use was associated with a 16% increased risk of stroke compared with non-users. The estimated effect was similar to the result of the meta-analysis of nine high-quality trials. Stratification analyses demonstrated that the PPI-associated absolute risk of stroke dramatically increased with the baseline predicted stroke risk. Additional analysis showed that H2RA, a less potent acid-suppressor, was not associated with stroke.

### Comparison with other studies

There are accumulating observational studies indicating an association between long-term use of PPIs and stroke [4–8, 10, 11]. However, these studies were generally limited by inadequate assessment of exposures and outcomes through retrospective recall or administrative claims, and insufficient adjustment of important confounders such as lifestyle habits and indications of PPI therapy. For example, a recent cohort of 214,998 individuals from Danish national registry data indicated that PPI use assessed by pharmacy records was associated with ischemic stroke (HR 1.13, 95% CI 1.09–1.19) [12]. However, lack of adjustment for several key risk factors for stroke, such as BMI, smoking status, and physical activity, and possible selection bias led to threats to the validity of the findings. Contrast with our results, another prospective analysis from the Nurses’ Health Study (NHS) and Health Professionals Follow-up Study (HPFS) found that regular PPI use was not associated with ischemic stroke after comprehensive adjustment for major known stroke risk factors (HR 1.08; 95% CI 0.91 to 1.27) [14]. The authors concluded that prior reports of an increased risk of stroke might be due to residual confounding related to chronic conditions associated with PPI use [14]. In the present analysis, we still observed a significant association after adjustment for indications of PPIs. A potential explanation for the inconsistency is that all the participants in the NHS and HPFS are health professionals who may have different characteristics from the UK Biobank cohort, which recruited general population. In addition, the sample size in the present study is much larger (0.492 million vs. 0.097 million), enabling us to detect the small increased risk.

In addition to the aforementioned observational studies, previous meta-analyses also demonstrated an increased risk of stroke in PPI users [11, 40]. In 2018, a meta-analysis of 12 RCTs and 10 cohort studies showed that concomitant use of PPI with thienopyridines was associated with an increased risk of ischemic stroke (RR 1.74, 95% CI 1.41-2.16) when compared with thienopyridines alone [11]. It should be noticed that although this meta-analysis included RCTs, most RCTs were not designed to specifically evaluate the effect of PPIs (the classification of the PPI group and the non-PPI group was not based on the randomization), so these RCTs should be considered as observational. Another meta-analysis of 15 articles and 155,406 patients found a positive association between the use of PPI and stroke (odds ratio [OR] 1.28, 95% CI 1.12–1.48) [40]. Subgroup analysis of RCTs did not find a significant increased risk (OR 1.47, 95% CI 0.66–3.25). However, this analysis only included 3 trials and further inspection of the original publications suggested that one study [41] was a cohort study with propensity score analysis which should not be considered as real randomized trial. Consistent with our results, two additional meta-analyses reported that PPI use was associated with an increased risk on the composite cardiovascular outcome (stroke, myocardial infarction, and cardiovascular death) [42, 43].

A large number of RCTs about PPIs have been published. Because our meta-analysis focused on stroke, only those trials with a relatively large sample size, long follow-up time, and comprehensive collection of outcomes, might have these data, and be included. These trials were usually well designed and strictly performed, so the methodological quality was high. We strictly evaluated the risk of bias based on the ROB 2 criteria; most included studies were at low risk of bias. Although two included trials were open, the impact was minor because stroke incidence was an objective endpoint.

Our results from the cohort showed that although the relative risks of stroke associated with PPI use were similar across different baseline stroke risk strata, the absolute risks increased with the baseline predicted risk. Similar findings were reported in the previous analyses of other medicines [20, 21]. For example, a cohort study reported that the absolute risk of cardiovascular events related to diclofenac increased progressively with baseline risk [20]. The risk of the onset of stroke associated with PPI use seems mainly concentrated among people with high baseline stroke risk, which could be easily evaluated by tools such as the Framingham Stroke Risk Score [26]. This finding established the evidence base for individualized use of PPIs.

### Possible mechanisms

The mechanism underlying the association between PPI use and stroke remains unclear. A possible pathway is that PPIs may increase the plasma asymmetric dimethylarginine (ADMA) level and reduce the nitric oxide (NO) [44, 45]. NO is an important vasoprotective molecule as it may reduce vascular cell proliferation, platelet adhesion and aggregation, and endothelial-leukocyte interactions [46]. Abnormalities in NO synthesis can cause endothelial dysfunction which, in turn, result in the development of various vascular diseases [46, 47]. PPIs have been demonstrated to increase ADMA levels by inhibiting the activity of dimethylarginine dimethylamnohydrolase (DDAH), which may competitively inhibits the nitric oxide enzyme [45, 48, 49]. In addition to inhibition of DDAH, it has been proposed that PPI use may prevent gastric NO formation from dietary nitrate and nitrite by suppressing gastric acidity [50]. Further, the previous study has shown that PPI use was associated with vitamin B12 deficiency [51], which could elevate homocysteine and subsequently increase ADMA levels, causing endothelial dysfunction [44]. In addition, it has also been shown that PPI use could result in diabetes [5] and metabolic syndrome [52], which in turn increase the risk of stroke.

### Limitations and strengths

This study lies in a well-established prospective cohort of over 0.49 million participants and 5182 events, providing sufficient statistical power to explore PPI-associated stroke and risk stratification. In addition, data about medication use were self-reported and verified by trained nurses, which might be more accurate to reflect the actual PPI use, including prescription and over-the-counter sources. More importantly, the availability of a wide range of demographic characteristics, lifestyle habits, comorbidities, drug use, and other covariates allowed us to comprehensively adjust for potential confounding effects. Our estimated effects were similar between cohort study and meta-analysis of RCTs, which provided the current best evidence for this association. The estimated effect of the meta-analysis was graded as the moderate quality of evidence based on the GRADE system. Our confidence in the meta-analysis was strengthened by the high overall methodological quality, low probability of publication bias, and robust sensitivity analyses.

This study has limitations. First, this study included participants with various underlying reasons for using PPIs, leading to potential confounding bias. However, the influence would be small because (1) adjustment for the indications for PPI use almost show no changes in the primary estimated effect, (2) subgroup analyses by indications show no evidence of interaction, (3) the estimated effects were similar to the meta-analysis of RCTs which is considered as with low confounding bias. Second, as the UK Biobank did not collect information on PPI use dosage, frequency, and duration, we could not explore the possible dose-response relationship. However, in our meta-analysis, different dosages (Table S1) were included and there was no heterogeneity, suggesting that the potential influence was minor. Third, although we carefully adjusted for a series of confounders in the cohort study, we could not completely rule out residual confounding effects. However, as the results were similar between the cohort and meta-analysis of RCTs, residual confounding effects, if any, would be minor. Fourth, reverse causality might exist. However, the results remained unchanged when excluding the cases identified during the first 2 years of follow-up. Fifth, we cannot be sure that participants used the prescribed medications during the follow-up and were unable to account for time-varying covariates, which might cause misclassifying exposure and immortal time bias. However, this bias seems more likely to drive associations to the null rather than to produce the positive associations we observed. Sixth, the COMPASS trial weighted 94.6% in the meta-analysis, however the influence to the primary estimated would be minor as (1) risk of this trial was low, (2) heterogeneity between this trial and others was low, and (3) sensitivity analysis by excluding this study show no major impact on the main results. Lastly, our meta-analysis was limited by insufficient data for the analysis of the stroke subtypes and a small number of events in most included trials.

## Conclusions

Overall, this prospective cohort study and meta-analysis of randomized trials indicated that regular PPI use was associated with an increased risk of stroke, with a higher net risk observed in individuals with high baseline stroke risk. Given the potential risk of stroke and other adverse effects such as enteric infections, fractures, and diabetes, [2–5] clinicians and patients should carefully balance the benefits and harms when using PPIs. An evaluation of baseline predicted stroke risk before prescription by widely used algorithms, such as Framingham Stroke Risk Score [26], is an efficient method to identify patients who may be associated with a high net risk of stroke. For high-risk population, carefully evaluating the need for long-term use of PPIs, seeking alternative therapeutic options, and routinely screening for stroke risk among those who have to take long-term PPI therapy are recommended. Future research, including well-designed cohort studies, larger-scale randomized controlled trials, and meta-analyses are required to confirm our conclusion. Basic science research to investigate the underlying mechanisms and clinical research on the optimal cut-off level of baseline stroke risk for individualized use of PPIs may further contribute to the rational use of this medicine.

## Supplementary Information

**Additional file 1:** The file contains additional analysis, such as sensitivity analysis and supplementary analysis; **Table S1.** Strategy for PubMed; **Table S2.** Baseline characteristics of included trials for meta-analyses; **Table S3.** Hazard ratio of stroke for individual class of proton pump inhibitors; **Table S4.** The risk of stroke associated with taking proton pump inhibitors according to clinical indication; **Table S5.** Sensitivity analyses of proton pump inhibitors and the risk of stroke; **Table S6.** Falsification analyses of proton pump inhibitor use and negative control outcomes; **Figure S1.** Estimated number needed to harm for regular PPI use and risk of stroke; **Figure S2.** Flowchart of study selection; **Figure S3.** Publication bias in the meta-analysis of proton-pump inhibitors and risk of stroke; STROBE Statement. Checklist of items that should be included in reports of cohort studies.

## Data Availability

The data that support the findings of this study are available from the UK Biobank (application number 51671, approved August 2019) but restrictions apply to the availability of these data, which were used under license for the current study. So the data are not publicly available. Data are however available from the authors upon reasonable request and with permission of UK Biobank. The UK Biobank is an open access resource.Researchers can apply to use the UK Biobank dataset by registering and applying at website (https://www.ukbiobank.ac.uk/). Data sets used for the analysis will be made available under reasonable requests.

## Abbreviations

ADMA: Asymmetric dimethylarginine
BMI: Body mass index
CI: Confidence interval
CVD: Cardiovascular disease
DDAH: Dimethylarginine dimethylamnohydrolase
GERD: Gastroesophageal reflux disease
HR: Hazard ratio
H2RAs: Histamine-2 receptor antagonists
ICD: International Classification of Diseases
NNH: Number needed to harm
NO: Nitric oxide
NSAIDs: Non-steroidal anti-inflammatory drugs
PPI: Proton pump inhibitor
RCT: Randomized controlled trial
RD: Risk difference
UK: United Kingdom
