# Supplementary material for: Regular use of proton-pump inhibitors and risk of stroke: a population-based cohort study and meta-analysis of randomized-controlled trials
Source: BMC Med. 2021 Dec 3;19:316. doi: 10.1186/s12916-021-02180-5 (PMC8641218; doi:10.1186/s12916-021-02180-5)
Supplement: Supplementary file 1 — Additional file 1: The file contains additional analysis, such as sensitivity analysis and supplementary analysis; Table S1. Strategy for PubMed; Table S2. Baseline characteristics of included trials for meta-analyses; Table S3. Hazard ratio of stroke for individual class of proton pump inhibitors; Table S4. The risk of stroke associated with taking proton pump inhibitors according to clinical indication; Table S5. Sensitivity analyses of proton pump inhibitors and the risk of stroke; Table S6. Falsification analyses of proton pump inhibitor use and negative control outcomes; Figure S1. Estimated number needed to harm for regular PPI use and risk of stroke; Figure S2. Flowchart of study selection; Figure S3. Publication bias in the meta-analysis of proton-pump inhibitors and risk of stroke; STROBE Statement. Checklist of items that should be included in reports of cohort studies. [file 12916_2021_2180_MOESM1_ESM.docx]

**Online Supplementary Materials**

**Table S1**. Strategy for PubMed

| **NO** | **Search terms** |
| --- | --- |
| 1 | Proton Pump Inhibitors/ |
| 2 | ((proton adj2 pump adj2 inhibitor$) or PPI or PPIs).ab. |
| 3 | Omeprazole/ |
| 4 | (omeprazole or losec or nexium or prilosec or rapinex or zegerid or ocid or Lomac or Omepral or Omez).ab. |
| 5 | Esomeprazole Sodium/ |
| 6 | (Esomeprazole or Nexium or Esotrex or Alenia or Escz or Esofag or Nexiam).ab. |
| 7 | (lansoprazole or lanzoprazole or agopton or bamalite or Inhibitol or Levant or Lupizole or lanzor or monolitum or ogast or ogastro or opiren or prevacid or prezal or pro ulco or promeco or takepron or ulpax or zoton).ab. |
| 8 | (rabeprazole or aciphex or dexrabeprazole or pariet or Zechin or Rabecid or Nzole‐D or Rabeloc).ab. |
| 9 | (Dexlansoprazole or Kapidex or Dexilant).ab. |
| 10 | (pantoprazole or protium or protonix or Pantotab or Pantopan or Pantozol or Pantor or Pantoloc or Astropan or Controloc or Pantecta or Inipomp or Somac or Pantodac or Zurcal or Zentro).ab. |
| 11 | or/1‐10 |
| 12 | randomized controlled trial.pt. |
| 13 | controlled clinical trial.pt. |
| 14 | randomized.ab. |
| 15 | placebo.ab. |
| 16 | placebo.fs. |
| 17 | randomly.ab. |
| 18 | trial.ab. |
| 19 | groups.ab. |
| 20 | or/12-19 |
| 21 | exp animals/ not humans.sh. |
| 22 | 20 not 21 |
| 23 | 11 and 22 |

**Table S2.** Baseline characteristics of included trials for meta-analyses.

| **Study** | **Location** | **Median age** | **Female (%)** | **Intervention (n)** | **Control (n)** | **Indication of PPI therapy** | **Follow-up** |
| --- | --- | --- | --- | --- | --- | --- | --- |
| Stupnicki 2003 [1] | Multinational trial | 64.0 | 376(73.0%) | Pantoprazole 20 mg (257) | Misoprostol 200 mg (258) | Prevention of NSAID-related gastrointestinal lesions | 6 months |
| Yeomans 2008 (ASTERIX) [2] | Multinational trial | 69.2 | 425(42.8%) | Esomeprazole 20 mg (493) | Placebo (498) | Prevention of low-dose aspirin-related GI lesions | 26 weeks |
| Bhatt 2010 (COGENT) [3] | Multinational trial | 68.6 | 2563(68.1%) | Omeprazole 20 mg (1876) | Placebo (1885) | Prevention of clopidogrel-related GI lesions | 6 months |
| Sugano 2011 [4] | Japan | 69.0 | 94(20.4%) | Lansoprazole 15 mg (226) | Placebo (235) | Prevention of low-dose aspirin-related GI lesions | 6 months |
| Hsu 2011 [5] | Taiwan | 72.0 | 42(24.8%) | Esomeprazole 20 mg +clopidogrel 75 mg (83); | Clopidogrel 75 mg (82) | Prevention of clopidogrel-related GI lesions | 6 months |
| Scheiman 2011 (OBERON) [6] | Multinational trial | 67.6 | 1269(47.7%) | Esomeprazole 40 mg (817); Esomeprazole 20 mg (804) | Placebo (805) | Prevention of low-dose aspirin-related GI lesions | 26 weeks |
| Sugano 2014 (LAVENDER) [7] | Multinational trial | 67.1 | 73(20.1%) | Esomeprazole 20 mg (182) | Placebo (182) | Prevention of low-dose aspirin-related GI lesions | 72 weeks |
| Attwood 2015 (SOPRAN) [8] | Multinational trial | 54.0 | 190(25%) | Omeprazole 20 or 40 mg (154) | Antireflux Surgery (144) | Reflux Esophagitis | 12 years |
| Moayyedi 2019 (COMPASS) [9] | Multinational trial | 67.7 | 3806(21.6%) | Pantoprazole 40 mg (8791) | Placebo (8807) | Prevention of rivaroxaban or aspirin-related GI lesions | 3 years |

**Table S3.** Hazard ratio of stroke for individual class of proton pump inhibitors

|  | **Person-years** | **HR [95% Cl]** |
| --- | --- | --- |
| **Type of PPIs** |  |  |
| No PPI use | 4326/3549337 | 1.00[Reference] |
| Omeprazole | 554/250245 | 1.18[1.06, 1.31] |
| Lansoprazole | 296/134738 | 1.09[0.96, 1.25] |
| Esomeprazole | 44/18940 | 1.16[0.84, 1.58] |
| Rabeprazole | 18/9040 | 0.93[0.58, 1.49] |
| Pantoprazole | 16/7512 | 1.03[0.62, 1.69] |

Abbreviation: CI, Confidence Interval; HR, Hazard Ratio; PPI, proton pump inhibitor; RD, risk difference.

Estimated effects were based on the fully adjusted model (see the footnote in Table 2).

| **Table S4.** The risk of stroke associated with taking proton pump inhibitors according to clinical indication | | | | | |
| --- | --- | --- | --- | --- | --- |
|  | Person-years | HR [95% Cl] | | | P-interaction |
|  |  | Age and gender-  stratified model | | Multivariable adjusted model |  |
| Any clinical indication for PPIs use |  | |  |  | 0.666 |
| No | 4499/3573880 | | 1.53[1.38, 1.69] | 1.17[1.06, 1.30] |  |
| Yes | 683/361150 | | 1.23[1.05, 1.44] | 1.11[0.94, 1.31] |  |

Abbreviation: CI, Confidence Interval; HR, Hazard Ratio.

Estimated effects were based on the fully adjusted model (see the footnote in Table 2).

| **Table S5.** Sensitivity analyses of proton pump inhibitors and the risk of stroke | | |
| --- | --- | --- |
|  | Cases/ Person-years | HR [95%CI] |
| **Lagging the exposure for 2 years to allow a time window for stroke incidence** | | |
| Non-regular PPI user | 3646/3546593 | 1.00[Reference] |
| Regular PPI user | 692/384984 | 1.12 [1.02, 1.24] |
| **Excluding the participants with cardiovascular disease at baseline** | | |
| Non-regular PPI user | 3800/3399294 | 1.00[Reference] |
| Regular PPI user | 600/325990 | 1.19 [1.07, 1.32] |
| **Excluding participants with cancer or cardiovascular disease at baseline** | | |
| Non-regular PPI user | 3498/3235695 | 1.00[Reference] |
| Regular PPI user | 531/302117 | 1.14 [1.04, 1.25] |
| **Excluding the participants with missing covariate data** | | |
| Non-regular PPI user | 3227/2743455 | 1.00[Reference] |
| Regular PPI user | 588/277770 | 1.17 [1.05, 1.30] |
| **Additionally adjusting for the** **patient health indicator variables** | | |
| Non-regular PPI user | 4326/3549337 | 1.00[Reference] |
| Regular PPI user | 856/385693 | 1.10 [1.01, 1.20] |
| **Using inverse treatment probability weighting based on the propensity score^*^** | | |
| Non-regular PPI user | 4326/3549337 | 1.00[Reference] |
| Regular PPI user | 856/385693 | 1.17 [1.13, 1.22] |

Abbreviation: CI, Confidence Interval; HR, Hazard Ratio.

Estimated effects were based on the fully adjusted model (see the footnote in Table 2).

| **Table S6.** Falsification analyses of proton pump inhibitor use and negative control outcomes | | |
| --- | --- | --- |
| **Outcome** | Cases/ Person-years | HR [95%CI] |
| **Malignant melanoma cancer** | |  |
| Non-regular PPI user | 1456/3187343 | 1.00[Reference] |
| Regular PPI user | 173/345815 | 1.00 [0.83, 1.20] |
| **Death caused by transportation** | |  |
| Non-regular PPI user | 64/3544833 | 1.00[Reference] |
| Regular PPI user | 8/397509 | 1.05[0.50, 2.20] |

Abbreviation: CI, Confidence Interval; HR, Hazard Ratio.

Estimated effects were based on the fully adjusted model (see the footnote in Table 2).

**
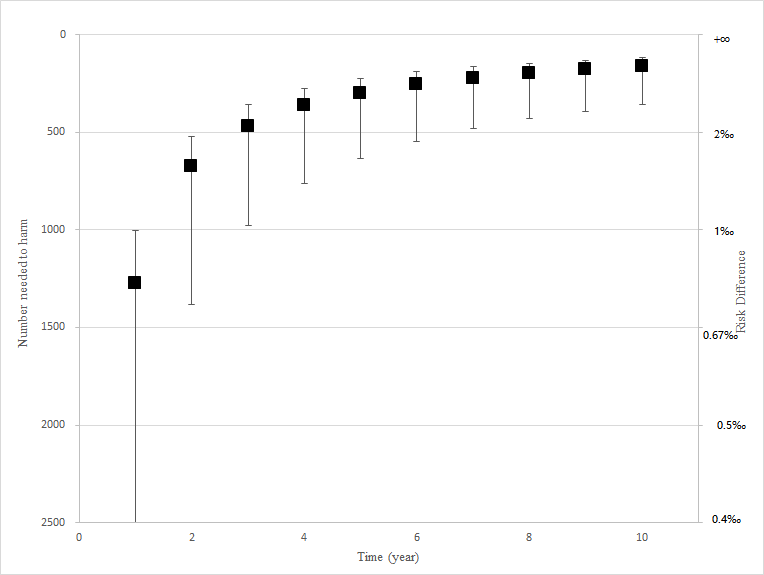
**

**Figure S1.** Estimated number needed to harm for regular PPI use and risk of stroke.

The estimated number needed to harm (NNT) was based on the fully adjusted hazard ratio of regular PPI use vs. non-use (1.16, 95% CI 1.06-1.27) and stroke rate in the non-use group (1.19 case/1000 person-years), with the method described by Altman D.G and Andersen P.K.


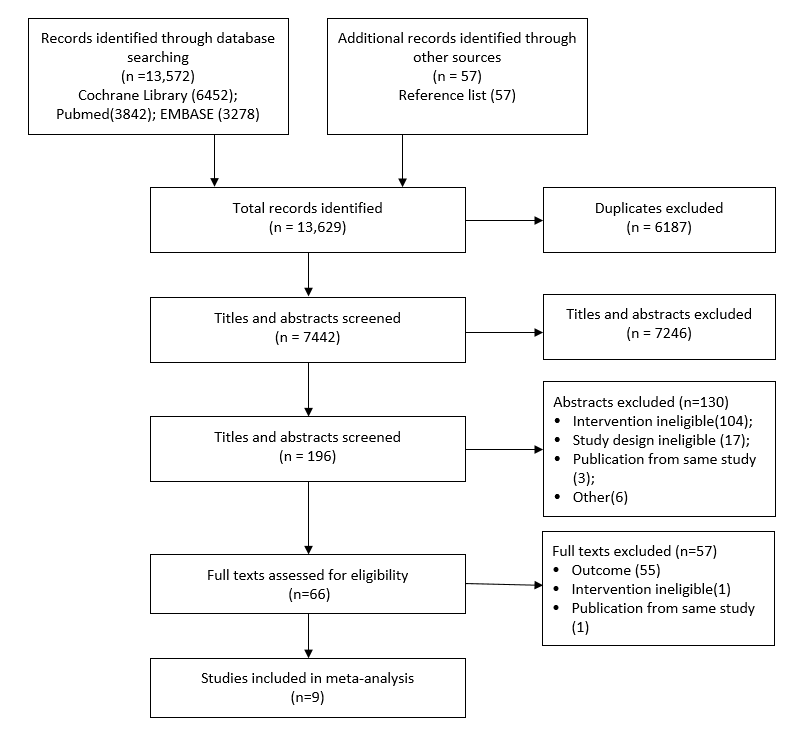


**Figure S2.** Flowchart of study selection


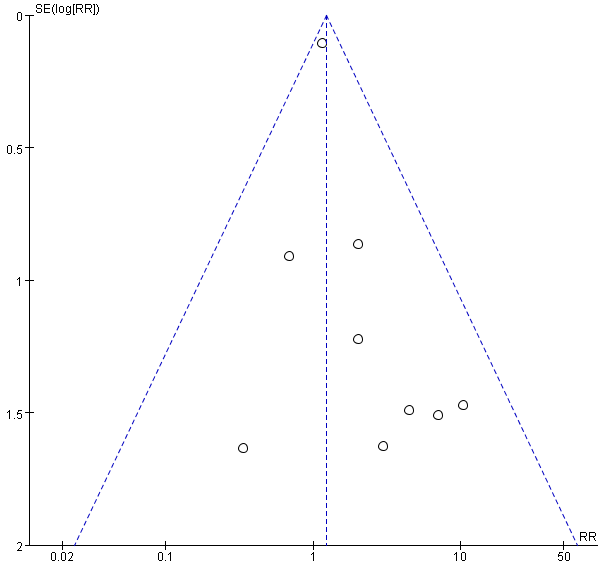


**Figure S3.** Publication bias in the meta-analysis of proton-pump inhibitors and risk of stroke.

Egger's test: P=0.19

STROBE Statement—Checklist of items that should be included in reports of ***cohort studies***

|  | Item No | Recommendation | Page No |
| --- | --- | --- | --- |
| **Title and abstract** | 1 | (*a*) Indicate the study’s design with a commonly used term in the title or the abstract | 1 |
|  |  | (*b*) Provide in the abstract an informative and balanced summary of what was done and what was found | 3 |
| Introduction | | | |
| Background/rationale | 2 | Explain the scientific background and rationale for the investigation being reported | 4 |
| Objectives | 3 | State specific objectives, including any prespecified hypotheses | 5 |
| Methods | | | |
| Study design | 4 | Present key elements of study design early in the paper | 5 |
| Setting | 5 | Describe the setting, locations, and relevant dates, including periods of recruitment, exposure, follow-up, and data collection | 6 |
| Participants | 6 | (*a*) Give the eligibility criteria, and the sources and methods of selection of participants. Describe methods of follow-up | 6 |
|  |  | (*b*) For matched studies, give matching criteria and number of exposed and unexposed | N.A. |
| Variables | 7 | Clearly define all outcomes, exposures, predictors, potential confounders, and effect modifiers. Give diagnostic criteria, if applicable | 6-7 |
| Data sources/ measurement | 8* | For each variable of interest, give sources of data and details of methods of assessment (measurement). Describe comparability of assessment methods if there is more than one group | 6-7 |
| Bias | 9 | Describe any efforts to address potential sources of bias | 7-8 |
| Study size | 10 | Explain how the study size was arrived at | N.A. |
| Quantitative variables | 11 | Explain how quantitative variables were handled in the analyses. If applicable, describe which groupings were chosen and why | 7-8 |
| Statistical methods | 12 | (*a*) Describe all statistical methods, including those used to control for confounding | 7-8 |
|  |  | (*b*) Describe any methods used to examine subgroups and interactions | 8 |
|  |  | (*c*) Explain how missing data were addressed | 8 |
|  |  | (*d*) If applicable, explain how loss to follow-up was addressed | 6 |
|  |  | (*e*) Describe any sensitivity analyses | 9 |
| Results | | |  |
| Participants | 13* | (a) Report numbers of individuals at each stage of study—eg numbers potentially eligible, examined for eligibility, confirmed eligible, included in the study, completing follow-up, and analysed | 9 |
|  |  | (b) Give reasons for non-participation at each stage | 9 |
|  |  | (c) Consider use of a flow diagram | Appendix |
| Descriptive data | 14* | (a) Give characteristics of study participants (eg demographic, clinical, social) and information on exposures and potential confounders |  |
|  |  | (b) Indicate number of participants with missing data for each variable of interest | 10 |
|  |  | (c) Summarise follow-up time (eg, average and total amount) |  |
| Outcome data | 15* | Report numbers of outcome events or summary measures over time | 10 |

| Main results | 16 | (*a*) Give unadjusted estimates and, if applicable, confounder-adjusted estimates and their precision (eg, 95% confidence interval). Make clear which confounders were adjusted for and why they were included | 10-12 |
| --- | --- | --- | --- |
|  |  | (*b*) Report category boundaries when continuous variables were categorized | N.A. |
|  |  | (*c*) If relevant, consider translating estimates of relative risk into absolute risk for a meaningful time period | 11-12 |
| Other analyses | 17 | Report other analyses done—eg analyses of subgroups and interactions, and sensitivity analyses | 12 |
| Discussion | | | |
| Key results | 18 | Summarise key results with reference to study objectives | 13 |
| Limitations | 19 | Discuss limitations of the study, taking into account sources of potential bias or imprecision. Discuss both direction and magnitude of any potential bias | 16-17 |
| Interpretation | 20 | Give a cautious overall interpretation of results considering objectives, limitations, multiplicity of analyses, results from similar studies, and other relevant evidence | 13-14 |
| Generalisability | 21 | Discuss the generalisability (external validity) of the study results | 16-17 |
| Other information | | | |
| Funding | 22 | Give the source of funding and the role of the funders for the present study and, if applicable, for the original study on which the present article is based | 18 |

*Give information separately for exposed and unexposed groups.

**Note:** An Explanation and Elaboration article discusses each checklist item and gives methodological background and published examples of transparent reporting. The STROBE checklist is best used in conjunction with this article (freely available on the Web sites of PLoS Medicine at http://www.plosmedicine.org/, Annals of Internal Medicine at http://www.annals.org/, and Epidemiology at http://www.epidem.com/). Information on the STROBE Initiative is available at http://www.strobe-statement.org.
